# Intentions to use contraceptives in Pakistan: implications for behavior change campaigns

**DOI:** 10.1186/1471-2458-10-450

**Published:** 2010-08-02

**Authors:** Sohail Agha

**Affiliations:** 1 Population Services International, 1120 19th Street, NW, Suite 600, Washington DC 20036. USA

## Abstract

**Background:**

Since 1990-91, traditional method use has increased at a faster rate in Pakistan than modern method use. The importance of hormonal methods or the IUD has diminished and that of traditional methods has increased in the method mix. There is a need to identify factors motivating and deterring the adoption of specific family planning methods among married men and women in Pakistan.

**Methods:**

In addition to social and demographic characteristics of respondents, a representative household survey collected information on psychological correlates of family planning behavior from 1,788 non-pregnant wives and 1,805 husbands with not-pregnant wives. Males and females were from separate households. Principal components analysis was conducted to identify the underlying constructs that were important for each gender. Multinomial logistic regression analysis was conducted to determine the correlates of male and female intentions to use contraceptive methods.

**Results:**

Amongst women, the perception that her in-laws support family planning use was the strongest determinant of her intentions to use contraceptive methods. A woman's belief in the importance of spacing children and her perception that a choice of methods and facilities with competent staff were available were also powerful drivers of her intentions to use contraceptive methods. The strongest obstacle to a woman's forming an intention to use contraceptive methods was her belief that family planning decisions were made by the husband and fertility was determined by God's will. Fears that family planning would harm a woman's womb lowered a woman's intentions to use methods requiring procedures, such as the IUD and female sterilization.

The perception that a responsible, caring, husband uses family planning to improve the standard of living of his family and to protect his wife's health was the most important determinant of a man's intention to use condoms. A husband's lack of self-efficacy in being able to discuss family planning with his wife was the strongest driver of the intention to use withdrawal. A man's fear that contraceptives would make a woman sterile and harm her womb lowered his intention to use modern contraceptive methods.

**Conclusions:**

These findings highlight the importance of having secondary target audiences such as mothers-in-law and husbands in family planning behavior change campaigns implemented in Pakistan. Campaigns that stress the importance of child spacing are likely to have an impact. Client perceptions of the quality of care are important determinants of intentions to use contraceptive methods in Pakistan. Client concerns that the IUD and sterilization procedures might harm a woman's womb and cause sterility should be addressed. The findings suggest that there is a need to assess the actual quality of service delivery in Pakistan.

## Background

There is growing consensus that a given behavior is more likely to occur if the intention to practice it is strong, if there are no environmental barriers to performing it, and if an individual has the skills and ability to perform the behavior [1-3]. Although intentions are conceptually very important, few studies have looked at predictors of contraceptive intentions [4]. A rare study re-interviewed married women from the 1992 Moroccan DHS in 1995 and examined the predictive effect of contraceptive use intentions on contraceptive use three years later. Reported contraceptive use intentions in 1992 had strong predictive effect on contraceptive use in 1995, even after controlling for a range of factors. The strength of this effect was second only to that of previous contraceptive use. Moreover, those women who reported intentions to use contraception in 1992 but were not using a method in 1995 had an unmet need for contraception [5].

The intention to perform a behavior is driven by the perceived costs of that behavior and the motivation to practice it. This is consistent with Easterlin's Synthesis Framework [6] in which the regulation of fertility is determined by the a) level of motivation to avoid pregnancy and b) the costs of regulating fertility (including both real and perceived social, psychological, health and monetary costs).

Previous studies that have examined barriers to contraceptive adoption in Pakistan have considered "family planning" as a general behavioral category without explicitly taking into account the possibility that the barriers and motivation to using specific methods may vary. When a strict definition of what constitutes a behavior is used (i.e. a behavior is an action with a target that occurs within a specific context and time period) [3], sterilization use is a different behavior than injectable use. For the development of behavior change campaigns, intervention planners need to know whether factors motivating the use of sterilization are similar or different from those motivating the use of a hormonal method. Previous studies have also not examined how gender influences the adoption of family planning methods in Pakistan. To design effective communications campaigns, planners should have an understanding of whether (and how) the perceived costs of using a method vary for men and women. If factors motivating men and women to adopt contraceptive methods are different, behavior change campaigns should target men and women through gender-specific messages.

**Figure 1:**
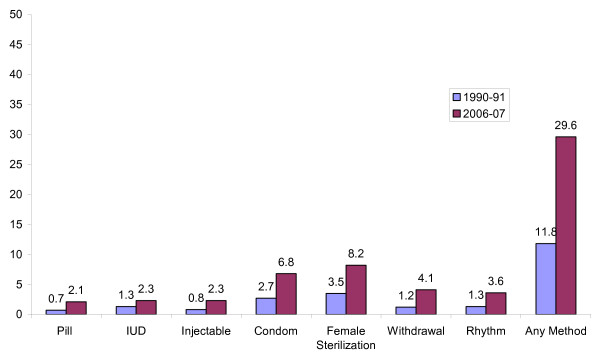
**Changes in contraceptive use in Pakistan**. Percentage of women that used a contraceptive method in Pakistan: 1990-91 and 2006-07 Pakistan Demographic and Health Surveys.

By assessing the perceived costs and motivations for specific contraceptive use behaviors, this study takes a step towards providing information which may help the development of a more effective behavior change communication strategy in Pakistan. With the exception of condoms and sterilization, there has been little increase in the use of modern contraceptive methods in Pakistan in the last 2 decades. Figure 1 shows Demographic and Health Surveys data on changes in use of contraceptive methods in Pakistan between 1990-91 and 2006-07. The use of the injectable, the pill and the IUD only increased from about 1% in 1990-91 to 2% in 2006-07. Condom use increased from 3% to 7%. The use of female sterilization increased from 3% to 8% [7,8]. Opposition to family planning by the husband or the wife and fear of method side-effects remain important barriers to contraceptive adoption [8]. Of note is the faster rate of increase of traditional methods of contraception than of modern methods of contraception in rural areas and the continued low share of modern female methods in the method mix in urban areas [9]. There is an urgent need to re-vitalize the Pakistan national family planning program. The findings of this study may contribute towards such an effort because of their implications for the marketing of contraceptive methods to married men and women in Pakistan.

### Literature Review

Contraceptive use trends in Pakistan during the last two decades raise concerns regarding the continued lack of effectiveness of the national family planning program. A recent study that compared changes in contraceptive use and the method mix in Pakistan using the 1990-91 and 2006-07 Demographic and Health Surveys, found a disturbing trend showing the declining importance of hormonal methods and the IUD in rural areas [9]. The share of traditional methods in the rural method mix increased from 17% to 26% during this period while the share of hormonal methods and the IUD declined from 33% to 26%. In urban areas, the share of hormonal methods and the IUD in the method mix remained stable during this period but remained relatively small at 18% [9]. Findings from this study highlight the importance of developing an understanding of factors that have increased the preference for traditional methods and reduced the importance of hormonal methods and the IUD in the method mix.

Studies that have examined the determinants of contraceptive use in Pakistan have looked at two main areas: a) the social and psychological costs of contraception and b) the contraceptive supply environment. Reporting on findings from the Pakistan IMPACT survey conducted in West Pakistan in 1968-69, Sirageldin found that while awareness of a family method was universal, there was limited knowledge of family planning personnel and facilities. Contraceptive prevalence was at 5.5%, with urban prevalence more than twice that of rural prevalence (9.8% vs. 3.9%). Visits to facilities which gave family planning advice were very low (5%) and were highly correlated with the use of family planning. Latent demand for family planning was high: 38% of urban wives and 31% of rural wives reported that their number of living children equaled or exceeded their ideal number and that they did not want any more children. When they examined intentions to use family planning in the future, the authors found that a woman's intentions to use family planning increased by 31% if her husband approved of family planning, and her intentions to use family planning were higher if a family planning facility was less than 30 minutes away. Sirageldin concluded that while latent demand for family planning existed, cultural and social constraints such as husband's approval prevented this demand from being converted into family planning use. The study indicated that the supply of information and services was too weak to catalyze latent demand into the use of family planning and that the program delivery system was seriously inadequate [10].

Besides Sirageldin [10], several studies have examined the influence of social and cultural factors on contraceptive use in Pakistan. These studies have emphasized the influence of the mother-in-law and the husband on family planning decision-making [11,12] and have highlighted the importance of communication between spouses regarding the use of contraception [2,13]. Fatalistic beliefs, particularly the belief that fertility is up to God, has also been found to have a powerful impact on the adoption of family planning [13]. This belief continues to have a powerful influence on the use of contraception: in 2006-07, 28% of Pakistani women who did not intend to use contraception in the future reported that fertility was determined by God's will [8].

One study [14], focused on identifying the range of social and psychological costs of family planning and their impact on Punjabi women's intentions to use family planning in the near future. Of the major obstacles to contraceptive adoption identified through a review of the literature, the study found that three of these obstacles had an important impact on intentions to use family planning: a woman's perception that contraceptive use would conflict with her husband's attitudes towards family planning or with his fertility preferences; her perception that contraceptives were socially or culturally unacceptable; her knowledge of contraception.

A limitation found in most studies that have examined the correlates of contraceptive adoption in Pakistan is that they have relied entirely on women's reports of obstacles to contraceptive adoption. The reliance on women's reports of barriers to contraceptive adoption and the virtual absence of published information on perceived obstacles to contraceptive adoption among men in Pakistan is a great limitation of the literature.

## Methods

### Study Design

The data for this analysis comes from the Pakistan Social Marketing Survey (PSMS) 2007. The PSMS is a nationally representative survey of currently married women 15-49 and men married to women 15-49. Men and women were selected from different households. The data for PSMS were collected by AcNielsen for Greenstar Social Marketing, with technical assistance from the Tulane University School of Public Health and Tropical Medicine. Ethics approval for the study was granted by the Tulane University Biomedical IRB. The survey was conducted in both urban and rural areas of all four provinces of Pakistan, with the exception of the Federally Administered Tribal Areas and the Federally Administered Northern Areas.

A multi-stage, stratified, sampling methodology was used. Urban areas were divided into four strata: those with population of more than five million, those with population between one and five million; those with population between 100,000 and one million; those with population less than 100,000. Rural areas were divided into three strata: those with population of more than 5,000; those with population between 3,000 and 5,000; those with population under 3,000.

#### Selection of cities and villages

The selection of cities/villages within each province and stratum was done by Probability Proportionate to Size sampling (PPS). Cities/villages were listed by population size and geographic order within strata and within provinces. The sampling interval was determined by dividing the population of these cities/villages by the number of cities/villages that were feasible to visit. The first city/village on the list was selected using a table of random numbers. Subsequent cities/villages were selected by adding the sampling interval to the cumulative population. This provided the requisite number of cities/villages within each stratum.

Twenty cities were selected in urban Pakistan. As per the Pakistan Census Organization, each city comprises of "charges" and "circles". The circle was the Primary Sampling Unit (PSU) in urban areas and 166 circles were randomly selected within these 20 cities. City maps were used to determine the boundaries of each circle. The circle was hypothetically divided into four quadrants and a random starting point was used to conduct 3 interviews in each quadrant. Twelve interviews were conducted within each circle (6 male and 6 female).

The village served as the PSU in rural areas. Two hundred villages were selected in rural Pakistan. Sketch maps were made of each village and each selected village was divided into four hypothetical quadrants. Using a random start, three interviews each were conducted from two quadrants and two interviews each from the remaining two quadrants. Ten interviews were conducted from each village (5 male and 5 female).

In both urban and rural areas, Field Supervisors physically visited the areas prior to data collection to get the lay of the land and select starting points for each quadrant. Starting points included mosques, schools, parks or markets. Starting points were allocated to interviewers prior to the initiation of data collection. They could not be changed once data collection began. Female interviewers used the Right Hand Rule to select households around a particular starting point. Male interviewers used the Left Hand Rule [15].

#### Selection of respondents from selected households

A Kish grid was used for the selection of an eligible respondent in each household. Only one respondent was interviewed per household. In the event that the selected respondent was not present, 3 attempts were made to reach the respondent. If the respondent could not be interviewed after 3 attempts, the household was dropped and the next household was selected using the random walk methodology.

#### Weighting of data

In total, 4,062 male and female respondents were interviewed. The sample was split evenly by gender and urban/rural residence. Weights were attached to the data to take the sampling strategy into account. The weights primarily adjusted the sample for representation of urban and rural areas as per the 1998 Census of Pakistan and for over-representation in Baluchistan. About 33% of Pakistan is urban. The weighted percentages reflect this proportion.

#### Instrument

The questionnaire included sections on socio-demographic characteristics of respondents, their reproductive intentions, their knowledge and use of family planning and their intentions to use contraceptive methods in the next 12 months. An important section of the questionnaire comprised of 35 statements regarding perceptions of the quality and availability of family planning services, norms about family planning, perceived social support for family planning, concerns about side effects of family planning and spousal support for family planning. Responses to these statements were on a five point scale, and went from strongly disagree to strongly agree. These attitudinal statements were originally developed for a reproductive health survey conducted in 2005 [16], after a review of the international literature on family planning. Modifications were made to the statements after they were pretested on a sample of 100 Pakistani respondents.

### Strengths and Limitations

A unique feature of the PSMS design is that both men and women were included in the survey. To our knowledge, the last publicly available nationally representative survey of Pakistan that included data on husbands was the Pakistan Demographic and Health Survey conducted in 1990-91. This study has many of the same limitations present in observational studies that make inferences based on cross sectional data.

### Variables and Statistical Analysis

#### Independent variables

Principal components analysis was conducted to identify the perceived costs of fertility regulation among married men and women in Pakistan. All factors with Eigenvalues greater than 1 were used for subsequent analysis (the Kaiser criterion). A varimax-rotated solution with eight factors produced the simplest structure for men: 1) responsible, caring husbands use family planning to improve the standard of living; 2) my in-laws support family planning; 3) I can easily obtain family planning methods or advice; 4) most people disapprove of family planning; 5) providers can be trusted to maintain confidentiality, to advise on method use and side-effects; 6) I am not able to discuss family planning with my spouse or to convince my spouse to use family planning; 7) family planning clinics have competent, friendly staff; 8) contraceptives can make a woman sterile, harm her womb, and are dangerous. Cumulatively, these 8 factors explained 65% of the total variance. See Additional file 1.

The varimax rotation identified seven factors for women: 1) child spacing protects a mother's health, caring spouses use family planning; 2) my in-laws support family planning; 3) I have access to choice of methods and facilities with competent providers; 4) providers can be trusted to maintain confidentiality, to advise on method use and side-effects; 5) I am not able to discuss family planning with my spouse or to convince my spouse to use family planning; 6) the husband decides if a wife can use family planning, God decides the number of children; 7) family planning can harm a woman's womb, modern methods can be very dangerous. Tables 1 and 2 in the appendix show results from the principal components analysis. Cumulatively, these 7 factors explained 66% of the total variance. See Additional file 2.

**Table 1:** Characteristics of married men whose wives are not pregnant and married non-pregnant women in the PSMS-1 sample

	Women (n = 1788)	Men (n = 1805)
**Province**		
Sindh	25.4%	25.4%
Punjab	55.7%	55.9%
Frontier	13.7%	14.0%
Baluchistan	5.3%	4.8%
		
**Urban**	32.9%	34.4%
		
Age***		
15-19	6.5%	1.0%
20-24	15.8%	9.3%
25-29	23.1%	15.0%
30-34	22.2%	16.9%
35-39	19.5%	18.6%
40-44	9.9%	16.4%
45-49	3.1%	11.9%
50 plus		10.8%
Number of living children**		
0-1	17.4%	21.8%
2-3	30.4%	26.0%
4-5	28.6%	28.9%
6 or more	23.6%	23.4%
Fertility desires**		
Want more/undecided	37.6%	42.1%
Do not want more children	62.4%	57.9%
Education***		
None	57.9%	31.1%
Primary	7.4%	8.0%
Middle	18.1%	30.4%
Secondary	9.8%	15.4%
Matriculate or higher	6.8%	15.1%
**Intend using a method in the next 12 months**		
Oral Contraceptive	5.1%	4.4%
IUD***	4.1%	1.6%
Injectable***	4.8%	2.5%
Condom**	11.3%	14.7%
Female Sterilization***	11.9%	7.6%
Withdrawal	6.5%	8.0%
Rhythm*	8.7%	6.6%
Any method***	41.7%	35.3%

*p < 0.05 **p < 0.01 ***p < 0.001

**Table 2:** Odds ratios (from a multinomial logistic regression) associated with intentions to use hormonal methods, the IUD, sterilization and traditional methods among women

	Intend using hormonal methods in next 12 months (n = 1788) (1)	Intend using the IUD in next 12 months (n = 1788) (2)	Intend using female sterilization in next 12 months (n = 1788) (3)	Intend using traditional methods in next 12 months (n = 1788) (4)
**Beliefs and perceptions**				
My in-laws support FP	2.12*** (1.68-2.68)	2.18*** (1.60-2.98)	2.19*** (1.78-2.70)	1.98*** (1.63-2.40)
Child-spacing protects mother's health, caring spouses use FP	2.05*** (1.59-2.64)	1.51** (1.11-2.06)	1.41** (1.14-1.75)	1.84*** (1.48-2.30)
I have access to choice of methods, and facilities with competent providers	1.82*** (1.48-2.25)	1.48** (1.13-1.95)	1.57*** (1.31-1.89)	1.63*** (1.37-1.94)
Providers can be trusted to maintain confidentiality, to advise on method use and side-effects	1.15 (0.94-1.40)	0.93 (0.73-1.19)	1.10 (0.93-1.31)	0.77** (0.65-0.90)
I am not able to discuss FP with spouse or convince spouse to use FP	0.83 (0.68-1.00)	0.95 (0.76-1.20)	0.73*** (0.62-0.86)	0.66*** (0.55-0.78)
Husband decides if wife can use family planning, God decides # of children	0.81* (0.66-0.99)	0.62*** (0.48-0.80)	0.64*** (0.54-0.77)	0.67*** (0.56-0.80)
Family planning can harm a woman's womb, modern method can be very dangerous	0.89 (0.73-1.08)	0.75* (0.59-0.96)	0.70*** (0.59-0.83)	0.93 (0.78-1.10)
				
**Province **(ref: Sindh)				
Punjab	0.99 (0.59-1.68)	1.55 (0.83-2.93)	1.54 (0.96-2.46)	2.92*** (1.76-4.85)
Frontier	2.13* (1.10-4.12)	0.88 (0.31-2.48)	0.56 (0.25-1.24)	3.56*** (1.83-6.92)
Baluchistan	3.62** (1.61-8.13)	0.42 (0.05-3.56)	0.88 (0.30-2.60)	1.59 (0.52-4.87)
				
**Urban **(ref: rural)	0.74 (0.46-1.19)	1.67 (0.96-2.93)	1.00 (0.66-1.51)	1.24 (0.85-1.81)
**Age **(ref: 35 plus)				
15-19	13.98*** (6.03-32.39)	4.78* (1.11-20.54)	0.79 (0.13-4.95)	1.69 (0.67-4.26)
20-24	8.39*** (4.23-16.62)	9.11*** (3.70-22.39)	1.01 (0.45-2.25)	2.74** (1.51-4.94)
25-29	5.55*** (3.01-10.26)	5.71*** (2.55-12.82)	1.78* (1.09-2.92)	2.65*** (1.60-4.40)
30-34	2.52** (1.36-4.66)	3.82*** (1.80-8.11)	0.94 (0.60-1.48)	2.34*** (1.48-3.69)
**Number of living children **(ref: 0-3)				
4-5	1.53 (0.92-2.53)	2.69** (1.39-5.21)	2.32** (1.44-3.74)	1.82** (1.18-2.81)
6 or more	2.09* (1.12-3.89)	4.81*** (2.06-11.22)	2.31** (1.36-3.91)	2.10** (1.23-3.58)
**Fertility desires **(ref: want more/undecided)				
Do not want more children	3.36*** (2.04-5.52)	1.49 (0.78-2.84)	27.93*** (9.12-85.54)	1.58* (1.02-2.45)
**Education **(ref: none)				
Primary	0.83 (0.37-1.86)	0.83 (0.27-2.60)	0.86 (0.44-1.68)	1.30 (0.70-2.39)
Middle	1.11 (0.65-1.90)	1.73 (0.87-3.44)	0.77 (0.46-1.30)	1.51 (0.97-2.37)
Secondary	1.00 (0.51-1.96)	3.02** (1.46-6.25)	0.78 (0.41-1.48)	1.12 (0.63-1.99)
Matriculate and higher	1.45 (0.60-3.50)	3.00* (1.13-7.94)	0.73 (0.29-1.83)	2.15* 1.10-4.20)
				
Pseudo R^2^	43.5%			

*p < 0.05 **p < 0.01 ***p < 0.001

Other independent variables primarily served the function of "control" variables. Previous studies have identified geographic (region, urban/rural) and socio-demographic variables (age, gender, education, number of children), and a proxy for motivation to regulate fertility (e.g. whether the respondent wanted to limit childbearing) as important determinants of contraceptive use in Pakistan [9,13,14,17].

#### Dependent variables

**Figure 2:**
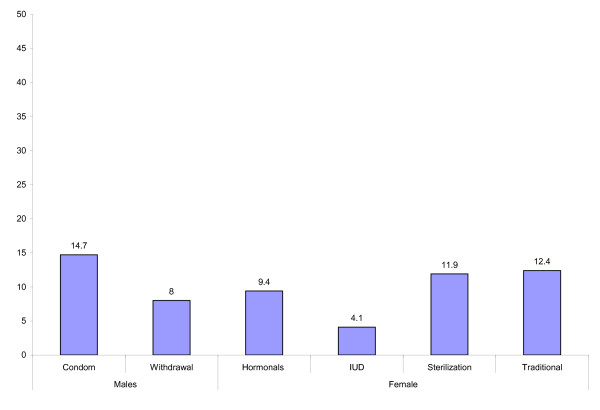
**Intentions to use contraceptive methods among married men and married women in Pakistan**. Percentage of married men and married women who intended to use contraceptive methods in the next 12 months: Pakistan Social Marketing Survey (PSMS) 2007.

All respondents in the sample, including currents users, were asked about intentions to use specific methods. Respondents were asked the following question: "Do you or your spouse intend to use this method in the next 12 months?" Respondents who reported an intention to use a method were coded as "1". All others were coded as "0". Figure 2 shows the percentage of married men and married women who intend to use contraceptive methods in the next 12 months. Intentions to use injectables and oral contraceptives were combined into one outcome variable. For the analysis of women's intentions, a six category dependent variable (no intentions, intentions to use hormonal methods, intentions to use the IUD, intentions to use female sterilization, intentions to use traditional methods and intentions to use condoms) was used in the multinomial logistic regression. For the analysis of men's intentions, a four category dependent variable (no intentions, intentions to use condoms, intentions to use withdrawal, intentions to use rhythm, and intentions to use modern methods besides condoms) was used in the multinomial logistic regression.

Analyses were also conducted with women who were currently not-using any contraceptive method. A binary outcome variable indicating non-users' intention to use any contraceptive method was used as the dependent variable for the logistic regression analyses.

## Results

Table 1 shows the characteristics of the sample of married women who were not pregnant at the time of the survey and of married men whose wives were not pregnant at the time of the survey. About a quarter of respondents were from Sindh, 56% from the Punjab, 14% from the Frontier and 5% from Baluchistan. About one-third of respondents lived in urban areas. Men were older than women, with 39% of men being 40 or older, compared to 13% of women. Although the mean number of living children was about the same (3.7 for males vs. 3.8 for females), a higher proportion of men had 0-1 children (22% vs. 17%). Women were more likely than men to not want any more children in the future (62% vs. 58%). Men had received more formal schooling than women: 58% of women and 31% of men had received no formal education.

Table 1 also shows intentions to use various contraceptive methods in the next 12 months. Males reported condoms (15%), withdrawal (8%) and female sterilization (8%) most frequently as the methods they or their spouses intended to use in the next 12 months. The methods females most commonly reported that they or their spouses intended to use in the next 12 months were female sterilization (12%), condoms (11%) and rhythm (9%). Overall, consistent with a higher level of desire to limit childbearing and a greater desire to better space pregnancies, women reported a higher level of intention to use a method in the next 12 months (42% vs. 35%) than men.

### Beliefs and perceptions driving women's intentions to use contraceptive methods

#### Hormonal methods (pill or the injectable)

Column 1 of Table 2 shows factors associated with a woman's intention to use a hormonal method. A woman's perceptions that her in-laws support family planning had the strongest impact on her intention to use a hormonal method (odds ratio = 2.12). The belief that child spacing protects a mother's health (odds ratio = 2.05) and the perception of having access to a choice of methods (odds ratio = 1.82) were also powerful predictors of hormonal method use. A woman's belief that her husband was the decision-maker regarding family planning use and that fertility was determined by God's will lowered her intention to use a hormonal method (odds ratio = 0.81).

#### The IUD

Column 2 of Table 2 shows factors associated with a woman's intention to use the IUD. The factors most strongly associated with a higher intention to use the IUD were perceived in-law's support (odds ratio = 2.18), belief in the health benefits of child spacing (odds ratio = 1.51) and perceived choice of methods (odds ratio = 1.48). A woman's belief that the use of family planning was the husband's decision was associated with a lower intention to use the IUD (odds ratio = 0.62). Fear that family planning could harm a woman's womb lowered a woman's intention to use the IUD (odds ratio = 0.75).

#### Female sterilization

Column 3 of Table 2 shows factors associated with a woman's intention to use female sterilization. Again, a woman's perception that her in-laws supported family planning had the strongest impact on the use of female sterilization (odds ratio = 2.19). Other factors such as the perception that a choice of methods and facilities with competent providers were available in her neighborhood (odds ratio = 1.57) were also important. Lack of self-efficacy in discussing family planning with her husband lowered a woman's intention to use female sterilization (odds ratio = 0.73). A woman's belief that the use of family planning was the husband's decision and that God decided the number of children a couple would have lowered her intention to use sterilization (odds ratio = 0.64). A woman's belief that family planning could harm a woman's womb lowered her intention to use female sterilization (odds ratio = 0.70).

#### Traditional methods

As with intentions to use hormonal methods and the IUD, a woman's perception that her in-laws supported family planning (odds ratio = 1.98) and her belief that child spacing protects a mother's health (odds ratio = 1.84) had the strongest effects on her intentions to use traditional methods. A woman's perception that providers could be trusted to maintain confidentiality, provide advice regarding side-effects of methods and their use, lowered her intention to use traditional methods (odds ratio = 0.77). A woman's perceived lack of self-efficacy in being able to discuss family planning with her spouse lowered her intention to use traditional methods (odds ratio = 0.66) as did her perception that the decision to use family planning fell in her husband's domain (odds ratio = 0.67).

### Control variables associated with women's intentions to use contraceptive methods

#### Region

Women in the Frontier (odds ratio = 2.13) and in Baluchistan (odds ratio = 3.62) were more likely to have an intention to use a hormonal method than women in Sindh. A recent national survey showed the popularity of hormonal methods was greater in the Frontier and Baluchistan provinces than in Sindh [8]. A woman in the Punjab (odds ratio = 2.92) or in the Frontier (odds ratio = 3.56) was more likely to have an intention to use a traditional method than a woman in Sindh. A recent national survey showed that the use of traditional methods was most popular among women in the Punjab [8].

#### Age and living children

Intentions to use hormonal methods, the IUD, or traditional methods were higher among women below age 35 compared to women 35 and older. Intentions to use contraceptive methods were higher among women with four or more children.

#### Desired fertility

A woman who wanted no more children was much more likely to intend using most contraceptive methods. The effect was particularly strong for sterilization (odds ratio = 27.93).

#### Education

Women with secondary (odds ratio = 3.02) or matriculate education (odds ratio = 3.00) had higher intentions to use the IUD. Women with matriculate or higher education also had a higher intention to use traditional methods (odds ratio = 2.15).

### Intention to use a contraceptive method among non-users of contraception

Table 3 shows factors associated with the intention to use a contraceptive method among women who were currently not using any contraceptive method. Overall, 10% of current non-users intended to use a contraceptive method in the next 12 months (not shown).

**Table 3:** Odds ratios (from a logistic regression) associated with intention to use a contraceptive method among women not currently using a contraceptive method

	Intend using a contraceptive method in next 12 months (n = 1045)
**Beliefs and perceptions**	
My in-laws support FP	1.99*** (1.59-2.47)
Child-spacing protects mother's health, caring spouses use FP	1.54*** (1.22-1.95)
I have access to choice of methods, and facilities with competent providers	1.35** (1.10-1.65)
Providers can be trusted to maintain confidentiality, to advise on method use and side-effects	0.96 (0.79-1.16)
I am not able to discuss FP with spouse or convince spouse to use FP	0.81* (0.67-0.98)
Husband decides if wife can use family planning, God decides # of children	0.74** (0.60-0.90)
Family planning can harm a woman's womb, modern method can be very dangerous	0.80* (0.65-0.98)
	
**Province **(ref: Sindh)	
Punjab	2.41** (1.40-4.17)
Frontier	2.07* (1.00-4.27)
Baluchistan	1.00 (0.27-3.76)
	
**Urban **(ref: rural)	1.20 (0.76-1.88)
	
**Age **(ref: 35 plus)	
15-19	5.01*** (2.25-11.15)
20-24	3.95*** (2.07-7.54)
25-29	3.03*** (1.70-5.42)
30-34	1.01 (0.54-1.90)
	
**Number of living children **(ref: 0-3)	
4-5	1.72* (1.03-2.88)
6 or more	1.52 (0.81-2.86)
	
**Fertility desires** (ref: want more or undecided)	
Do not want more children	2.42** (1.46-4.00)
	
**Education **(ref: none)	
Primary	1.60 (0.85-3.01)
Middle	0.90 (0.52-1.57)
Secondary	0.99 (0.49-2.00)
Matriculate and higher	1.86 (0.83-4.15)
Pseudo R^2^	22.2%

*p < 0.05 **p < 0.01 ***p < 0.001

The most powerful determinant of the intention to use a method among current non-users was a woman's perception that her in-laws supported family planning (odds ratio = 1.99), followed by the belief that child spacing protects a mother's health (odds ratio = 1.54). A woman's perception of having access to a choice of methods and competent providers increased her intention to use a method in the next 12 months (odds ratio = 1.35).

Obstacles to a woman's forming an intention to use a method included her inability to discuss family planning with her spouse (odds ratio = 0.81), her perception that the husband was the decision-maker regarding family planning and that God determined the number of children (odds ratio = 0.74) and her perception that using family planning posed a risk to her health (odds ratio = 0.80).

Non-users of a contraceptive method in the Punjab and the Frontier were more likely to intend using a contraceptive method in the next 12 months. Non-users under 30 years of age were more likely to intend using a method than women aged 35 or older. Non-users with four or five children were more likely to intend using a contraceptive method, as were non-users who wanted to limit childbearing.

### Beliefs and perceptions driving men's intentions to use contraceptive methods

#### Condoms

Column 1 of Table 4 shows factors associated with a man's intention to use condoms in the next 12 months. The variable with the strongest impact on a man's intentions to use condoms was the belief that a responsible and caring husband used family planning to improve the standard of living of the family and protect his wife's health (odds ratio = 1.55). A man who felt that he could easily obtain a family planning method or advice was more likely to have the intention to use a condom (odds ratio = 1.34). A man's perception that his in-laws support the use of family planning increased the likelihood of his using condoms (odds ratio = 1.21). A man's lack of self-efficacy in discussing family planning with his wife and his inability to convince his wife to use family planning was associated with a lower intention to use a condom (odds ratio = 0.82).

**Table 4:** Odds ratios (from a multinomial logistic regression) associated with intentions to use condoms, withdrawal and other modern methods among men

	Intend using condoms in next 12 months (n = 1805) (1)	Intend using withdrawal in next 12 months (n = 1805) (2)	Intend using any other modern method in next 12 months (n = 1805) (3)
**Beliefs and perceptions**			
My in-laws support FP	1.21* (1.02-1.43)	0.70** (0.57-0.86)	1.37** (1.14-1.64)
Responsible, caring husbands use FP to improve standard of living and protect mother's health	1.55*** (1.29-1.86)	0.90 (0.74-1.10)	1.63*** (1.34-2.00)
I can easily obtain FP methods or advice	1.34** (1.13-1.59)	1.29* (1.03-1.62)	1.20* (1.01-1.42)
Providers can be trusted to maintain confidentiality, to advise on method use and side-effects	0.99 (0.85-1.16)	1.04 (0.84-1.27)	1.12 (0.95-1.31)
FP clinics have competent, friendly staff	1.19* (1.01-1.41)	1.47** (1.16-1.86)	1.16 (0.99-1.35)
I am not able to discuss FP with spouse or convince spouse to use FP	0.82* (0.70-0.96)	1.51*** (1.23-1.84)	0.93 (0.78-1.10)
Most people disapprove of FP	0.97 (0.83-1.14)	0.94 (0.77-1.14)	1.09 (0.93-1.29)
Contraceptives can make a woman sterile, harm her womb, are dangerous	0.94 (0.80-1.09)	1.11 (0.91-1.36)	0.81** (0.68-0.95)
**Province **(ref: Sindh)			
Punjab	0.90 (0.60-1.36)	0.60* (0.37-0.96)	1.43 (0.90-2.26)
Frontier	6.30*** (3.62-10.89)	1.57 (0.84-2.93)	2.87*** (1.48-5.58)
Baluchistan	0.70 (0.22-2.21)	1.10 (0.46-2.66)	1.88 (0.63-5.62)
			
**Urban **(ref: rural)	2.15*** (1.52-3.05)	1.87** (1.21-2.88)	1.14 (0.78-1.65)
**Age **(ref: 45 plus)			
15-24	2.02* (1.00-4.08)	0.91 (0.34-2.45)	1.46 (0.55-3.87)
25-29	2.36** (1.30-4.29)	1.32 (0.62-2.82)	4.96*** (2.66-9.23)
30-34	2.96*** (1.74-5.03)	2.90*** (1.64-5.13)	2.69** (1.54-4.70)
35-39	2.62*** (1.57-4.36)	1.94* (1.10-3.42)	2.25** (1.35-3.76)
40-44	2.18** (1.28-3.72)	1.19 (0.65-2.18)	2.45*** (1.49-4.02)
**Number of living children **(ref: 0-3)			
4-5	0.80 (0.54-1.20)	1.17 (0.72-1.89)	1.63* (1.05-2.53)
6 or more	0.47** (0.28-0.77)	0.81 (0.46-1.44)	1.14 (0.70-1.87)
**Fertility desires** (ref: want more children or undecided)			
Do not want more children	2.54*** (1.73-3.72)	3.21*** (1.98-5.18)	7.78*** (4.68-12.92)
**Education **(ref: none)			
Primary	0.57 (0.25-1.29)	1.37 (0.61-3.10)	1.13 (0.62-2.06)
Middle	1.03 (0.68-1.56)	1.74* (1.01-2.99)	0.79 (0.53-1.19)
Secondary	1.50 (0.94-2.38)	2.42** (1.34-4.36)	0.81 (0.48-1.38)
Matriculate and higher	1.45 (0.90-2.34)	2.80** (1.55-5.05)	0.75 (0.43-1.33)
Pseudo R^2^	33.1%		

*p < 0.05 **p < 0.01 ***p < 0.001

#### Withdrawal

Column 2 of Table 4 shows that a man's perception that his in-laws supported family planning use lowered the likelihood of his using withdrawal (odds ration = 0.70), possibly because in-laws' support increased the likelihood of his using condoms or of his wife using other modern methods. A man's lack of self-efficacy in discussing family planning with his wife or in being able to convince her to use family planning had the strongest impact on the intention to use withdrawal: a man who was unable to discuss family planning with his spouse or unable to convince her to use family planning was more likely to use withdrawal (odds ratio = 1.51).

#### Modern methods other than the condom

Column 3 of Table 4 shows that the strongest predictor of a man's intention to use a modern method besides the condom was the perceived role of the husband: a man who believed that a responsible, caring husband used family planning was more likely to use a modern method (odds ration = 1.63). In-laws' support of family planning was another strong predictor of the intention to use a modern method other than a condom (odds ratio = 1.37). The perceived availability of family planning methods or advice (odds ratio = 1.20) was also associated with higher intentions to use a modern method other than a condom. The fear that contraceptives could make a woman sterile and harm her lowered a man's intention to use a modern method other than a condom (odds ration = 0.81).

### Control variables associated with a man's intentions to use a modern method other than the condom

#### Region

Residence in the Frontier province was associated with higher intentions to use condoms (odds ratio = 6.30) and other modern methods (odds ratio = 2.87).

#### Urban residence

Urban residence increased the likelihood of a man's having the intention to use a condom (odds ratio = 2.15) and the intention to use withdrawal (odds ratio = 1.87).

#### Age and living children

Compared to men 45 and older, the intention to use condoms as well as other modern methods was higher among those under age 45. Men with six or more children were less likely to intend using a condom than men with three or fewer children (odds ratio = 0.47). Men with four or five children were more likely to use a modern method other than a condom than men with three or fewer children (odds ratio = 1.63).

#### Desired fertility

The desire to limit childbearing was associated with higher intentions to use condoms (odds ratio = 2.54), higher intentions to use withdrawal (odds ratio = 3.21) and higher intentions to use a modern method other than a condom (odds ratio = 7.78).

#### Education

A man's education was associated with a higher intention to use withdrawal. Men with middle (odds ratio = 1.74), secondary (odds ratio = 2.42) or matriculate and higher (odds ratio = 2.80) education were more likely to use withdrawal than men with no education.

## Discussion

The findings of this study show that the psychosocial factors that influence men and women's intentions to use family planning methods are different. The stronger a man's sense that family planning use is a responsible behavior because it improves his wife's health and his family's economic well-being, the more likely he is to intend using a condom or another modern contraceptive methods. Interventions that appeal to a man's sense of responsibility towards his family are likely to motivate men to use modern contraceptive methods. Such a strategy may be particularly important in rural Pakistan, where a recent study found a much faster growth of traditional methods than of modern methods [9].

Men who were able to discuss family planning with their wives were more likely to have an intention to use a condom. These findings suggest that a decision to use the male condom is not a unilateral decision: some married men face opposition to family planning use from their wives and their ability to discuss contraception with their wives and to convince them to use contraceptive methods helps them to implement modern method use. When they are unable to discuss family planning with their wives, men are more likely to use withdrawal - a less effective method than the condom. Much of the previous literature on Pakistan emphasizes the role of the husband as an obstacle to family planning use by their wives. The situation is actually more complex. The findings of this study show that wives who are not open to discussing family planning can prevent their husbands from forming an intention to use family planning.

The findings also suggest that Pakistani women are more reliant on their husbands for the use of certain family planning methods than others. A woman was more likely to form an intention to use sterilization or to form an intention to use a traditional method if she was able to discuss family planning use with her husband. Both these methods require the husband's cooperation: in Pakistan, the protocol for providing a female sterilization requires a woman's getting explicit permission from her husband; rhythm can only be used as a family planning method if the husband is willing to abstain from intercourse during a woman's fertile period; withdrawal also needs the husband's active participation.

Independent of other factors, a woman who felt that decision-making regarding family planning was her husband's domain or that the number of children that a person had was decided by God was less likely to form an intention to use modern or traditional contraceptive methods. Men should also be considered a secondary target audience when contraceptive methods are being marketed to women. These findings are consistent with findings from the 2006-07 Demographic and Health Survey regarding reasons for non-use among women who do not intend to use contraception in the future: the two most important reasons for future non-use of contraception were that fertility was up to God (28%) and the husband was opposed to family planning use (10%) [8]. A Pakistani woman's lack of perceived control over decision-making regarding family planning highlights the importance of persuading Pakistani husbands to use contraception and to discuss contraceptive use with their wives.

A married woman's belief in the benefits of spacing children for a mother's health was a powerful predictor of her intention to use a hormonal method or an IUD. Historically, the national family planning program in Pakistan has focused on family planning as a way of limiting childbearing, not as a way of spacing births. It is extremely important for behavior change campaigns to stress the health benefits of spacing.

A woman's perception that her in-laws support family planning use is important for the formation of her intention to use a contraceptive method. This variable had a consistent, positive impact on the intention to use contraceptive methods. Managers of communications campaigns should consider directly targeting mothers-in-law to increase their support for the use of family planning. Such a strategy is likely to result in an increase in intentions to use contraceptives among reproductive age women.

A woman's perception of quality of care includes having a choice available in terms of methods and having access to competent providers. Perceived quality of care is an important driver of the intention to use hormonal methods, the IUD and female sterilization. With the declining importance of hormonal methods and the IUD in the method mix in Pakistan [9], the need to increase the availability of high quality family planning services cannot be underestimated.

Women with concerns that family planning use can harm their womb are less likely to have an intention to use sterilization. Campaigns to increase sterilization use should communicate the message that sterilization does not harm a woman's womb. Exploratory research to find out about women's perceptions regarding how sterilization impacts their bodies may be extremely useful in developing persuasive messages. At the same time, some assessment is needed of the quality of services provided for procedures such as IUD insertions and sterilization to ensure that women's fears regarding the potential harm that IUD insertion or sterilization can cause are indeed unfounded. In the 2006-07 Pakistan Demographic and Health Survey, about 10% of women not intending to use contraceptives in the future reported health concerns or side-effects as the reason for not intending to use contraception [8].

The drivers of and the barriers to having the intention to use contraception among non-users were consistent with the findings from all women: in-laws' support of family planning was the most powerful driver of intentions to use a contraceptive method, followed by the belief that child spacing protects a mother's health, and a woman's perception of having access to methods and competent providers. A woman's lack of self-efficacy in being able to discuss family planning with her husband, her belief that a husband makes the decision regarding family planning use, and the perception that family planning could harm a woman's womb were obstacles to the formation of an intention to use a method.

This focus of this study has been on factors associated with the intentions to use contraceptives. One recent Pakistani study using panel data has shown a powerful relationship between intentions to use condoms and condom use in a subsequent time period [18]. Intentions to perform a behavior translate into actual behavior when environmental obstacles can be overcome and individuals have skills to perform the behaviors [1-3]. Poor quality of service delivery is an important environmental barrier that may need to be overcome before substantial levels of adoption of female contraceptive methods occur in Pakistan.

With the exception of a few recent studies [18,19], there is a near-absence of assessments of the success or failure of strategies adopted to change family planning behavior in Pakistan. Much better understanding and documentation regarding the types of interventions that have had success in Pakistan is needed. In the absence of assessments of successes and failures of specific interventions, it will not be possible to make good programmatic investments in the future. The failure of Pakistan's family planning program to motivate couples with unmet need who have been reached by the program but not convinced to use contraception [13] calls for renewed efforts to launch behavior change campaigns supported by strong service delivery initiatives and impact evaluations.

## Conclusions

This study has identified a range of psychosocial factors that influence male and female intentions to use family planning methods. Behavior change campaigns implemented in Pakistan should address the drivers of contraceptive adoption identified in this study and lower the psychosocial costs associated with method use.

At the same time, there is a need to address quality of care issues surrounding the provision of family planning services in Pakistan. There is a near-absence of data on the actual quality of care provided to women seeking family planning services. Given the declining importance of hormonal methods and the IUD in the method mix in Pakistan, and women's perceptions of poor quality being a barrier to contraceptive adoption much in the same way as was the case four decades ago [10], urgent attention needs to be given to determine what actually is the level of quality of care being provided and how it can be improved.

## Competing interests

The author declares that they have no competing interests.

## Authors' contributions

SA was responsible for the design of the study, the data analysis and the write-up.

## Pre-publication history

The pre-publication history for this paper can be accessed here:

http://www.biomedcentral.com/1471-2458/10/450/prepub

## Supplementary Material

Additional file 1 **Rotated Component-Matrix - Men**. Click here for file

Additional file 2 **Rotated Component-Matrix - Women**. Click here for file
