# Supplementary material for: Intentions to use contraceptives in Pakistan: implications for behavior change campaigns
Source: BMC Public Health. 2010 Aug 2;10:450. doi: 10.1186/1471-2458-10-450 (PMC2920282; doi:10.1186/1471-2458-10-450)
Supplement: Additional file 2 — Rotated Component-Matrix - Women. [file 1471-2458-10-450-S2.DOC]

Additional file 2: Rotated Component-Matrix - Women

|  | 1 | 2 | 3 | 4 | 5 | 6 | 7 |
| --- | --- | --- | --- | --- | --- | --- | --- |
| Child spacing protects the health of the mother | 0.828 |  |  |  |  |  |  |
| Spouses who care for one another will use family planning | 0.813 |  |  |  |  |  |  |
| Family planning can help improve one’s standard of living | 0.806 |  |  |  |  |  |  |
| Men should share the responsibility of family planning | 0.780 |  |  |  |  |  |  |
| If I decided to use FP my in-laws would support me |  | 0.889 |  |  |  |  |  |
| If I used FP and had problems, in-laws would support me |  | 0.876 |  |  |  |  |  |
| My friends are all positive about the use of FP |  | 0.761 |  |  |  |  |  |
| My parents would support my decision regarding childbearing |  | 0.742 |  |  |  |  |  |
| There are many types of FP methods available in this area |  |  | 0.878 |  |  |  |  |
| There is clinic where I could get FP advice in neighborhood |  |  | 0.875 |  |  |  |  |
| Family planning services have knowledgeable staff |  |  | 0.845 |  |  |  |  |
| I know a place nearby where I can obtain contraceptives |  |  | 0.837 |  |  |  |  |
| Medical staff at FP clinics around here is helpful & friendly |  |  | 0.835 |  |  |  |  |
| FP clinics in this area have doctors available |  |  | 0.771 |  |  |  |  |
| I can easily obtain the FP method that I want to use |  |  | 0.751 |  |  |  |  |
| FP products are usually of good quality |  |  | 0.680 |  |  |  |  |
| Providers give good advice on the use of methods |  |  | 0.643 | 0.563 |  |  |  |
| You can trust FP providers to keep questions confidential |  |  | 0.632 | 0.510 |  |  |  |
| Providers advise on how to deal with method side-effects |  |  | 0.602 | 0.574 |  |  |  |
| FP methods are expensive |  |  | 0.473 |  | 0.365 |  |  |
| I am hesitant to discuss FP with my spouse |  |  |  |  | 0.726 |  |  |
| If my spouse opposes FP use, I am unable to convince him |  |  |  |  | 0.715 |  |  |
| There is nothing I can do about getting pregnant too soon |  |  |  |  | 0.711 |  |  |
| FP should only be used by older women who don’t want children |  |  |  |  | 0.658 | 0.330 |  |
| FP not considered a good thing among most people I know |  |  |  |  | 0.647 | 0.322 |  |
| Among people I know, FP is done secretly |  |  |  | -0.338 | 0.488 |  |  |
| For most couples, husband decides whether wife can use FP |  |  |  |  | 0.439 | 0.633 |  |
| Only God determines the number of children a couple has | 0.365 |  |  |  |  | 0.596 |  |
| Contraceptives can harm your womb |  |  |  |  |  |  | 0.824 |
| Modern contraceptives are very dangerous for a woman’s health |  |  |  |  |  |  | 0.819 |
| Contraceptives can make you sterile |  |  |  |  |  |  | 0.799 |
| Contraceptives are not effective in preventing pregnancy |  |  |  |  |  |  | 0.741 |
| Using some contraceptives can lead to side-effects |  |  |  |  |  |  | 0.740 |
| Lots of rumors that make me wonder if contraceptives work |  |  |  |  |  |  | 0.732 |
| Woman using contraceptives won’t be able to get pregnant |  |  |  |  |  |  | 0.619 |
